# Preoperative Neutrophil-to-Lymphocyte Ratio as a Prognostic Factor in Uterine Sarcoma

**DOI:** 10.3390/jcm9092898

**Published:** 2020-09-08

**Authors:** Min Jin Jeong, Jung Hyun Park, Soo Young Hur, Chan Joo Kim, Hae Seong Nam, Yong Seok Lee

**Affiliations:** 1 Department of Obstetrics and Gynecology, College of Medicine, The Catholic University of Korea, Seoul 03312, Korea; mjobgy@catholic.ac.kr (M.J.J.); jung0229@gmail.com (J.H.P.); hursy@catholic.ac.kr (S.Y.H.); chanjoo@catholic.ac.kr (C.J.K.); 2 Division of Pulmonology, Department of Internal Medicine, Inha University Hospital, Inha University, School of Medicine, Incheon 22332, Korea

**Keywords:** neutrophil-to-lymphocyte ratio, uterine sarcoma, prognostic marker, systemic inflammation marker, tumor microenvironment

## Abstract

Background: Recent studies have demonstrated that the tumor microenvironment, known to be influenced by inflammatory cells, plays a crucial role in cancer progression and clinical outcome of patients. The objective of the present study was to investigate prognostic values of preoperative neutrophil-to-lymphocyte ratio (NLR) and platelet-to-lymphocyte ratio (PLR) for disease-free survival (DFS) and overall survival (OS) of uterine sarcoma patients. Methods: Ninety-nine patients with uterine sarcoma treated in eight multicenter institutions over the last 20 years were retrospectively analyzed. Curves of DFS and OS were calculated using the Kaplan–Meier method, and univariate and multivariate analyses of various prognostic factors were performed using a Cox proportional hazard regression model. Results: High NLR was significantly associated with worse DFS (*p* = 0.007) and OS (*p* = 0.039). Advanced stage (*p* = 0.017) and high mitotic index (*p* = 0.036) retained their prognostic significance for DFS. Other clinical variables, including PLR, CA125, and lactate dehydrogenase (LDH) failed to show significant impact. Conclusions: Our findings showed that an elevated preoperative NLR was associated with poor clinical outcome in uterine sarcoma patients. Our results suggest that high NLR in early-stage uterine sarcoma patients might indicate that such patients need more intensive treatments.

## 1. Introduction

Uterine sarcoma is a rare gynecologic tumor, accounting for 1–3% of all female genital tract malignancies. Its incidence is about 3–5% of primary uterine malignancies [1]. Uterine sarcoma might originate from the smooth muscle in myometrium (leiomyosarcoma (LMS)), from the endometrial stroma (endometrial stromal sarcoma (ESS) and undifferentiated endometrial sarcoma (UES)), or from both (adenosarcoma) [2]. The International Federation of Gynecology and Obstetrics (FIGO) developed a new staging system for uterine sarcomas in 2009. Carcinosarcoma is no longer considered as part of uterine sarcomas. It is classified as a dedifferentiated or metaplastic form of endometrial carcinoma. Although the aggressive behavior of uterine sarcoma is well recognized, the rarity of this tumor and histopathological diversity of this disease make it difficult to establish appropriate diagnosis and treatment. Actually, the main problem in the management of uterine sarcoma is the notable absence of data from randomized trials concerning surgical and adjuvant treatments. Similarly, there are no markers to help diagnose uterine sarcoma or predict prognosis, making it more problematic to plan treatment [3]. Few studies have suggested that serum CA-125 and lactate dehydrogenase (LDH) levels might be helpful for preoperative diagnosis and surveillance of uterine sarcomas. However, their efficacy remains controversial [4,5].

The dynamic interplay between neoplastic cells and the immune microenvironment regulates every step of the metastatic process. Neutrophils play major roles linking inflammation and cancer and are actively involved in progression and metastasis [6]. High neutrophil-to-lymphocyte ratio (NLR) and platelet-to-lymphocyte ratio (PLR) have been found to be associated with poor survival rates of patients with the majority of solid tumors, including ovarian cancer [7,8,9]. Additionally, recent data suggest that neutrophils could be considered one of the emerging targets for multiple cancer types [10]. However, only a few studies have investigated such associations in patients with uterine sarcoma [11,12]. Thus, the aim of this study was to estimate prognostic values of NLR, PLR, and other clinical variables in uterine sarcoma patients after curative surgery.

## 2. Materials and Methods

### 2.1. Subjects

The Catholic University of Korea implemented a clinical data warehouse of data for ~2.8 million patients, 47 million prescription events, and laboratory results for 150 million cases. Clinical data (including personal data, laboratory data, diagnoses, medications, and operation records) of patients with uterine sarcoma who underwent surgical resection at the Department of Gynecologic Oncology at Seoul St. Mary’s Hospital and seven branch hospitals (Eunpyeong, Yeouido, Uijeongbu, Incheon, Bucheon St. Mary’s Hospital, St. Vincent, and St. Paul’s Hospital) from 1 January 2000 to 30 June 2020 were collected. All files of extracted data were encoded to prevent personal identification of patients during the sorting process. All patients who were diagnosed with uterine sarcoma, including LMS, ESS, undifferentiated sarcoma, and adenosarcoma, were retrospectively reviewed. Eight patients who had no follow-up data after surgery and one patient who was diagnosed with uterine sarcoma and blood cancer were excluded. Carcinosarcoma has been newly classified as a dedifferentiated or metaplastic form of endometrial carcinoma. Thus, those who had carcinosarcoma were also excluded from this study. The remaining 99 patients were included as the study population. Cancer stages were re-assessed for all patients according to FIGO 2018 staging system. Preoperative NLR and PLR were measured from routinely performed blood count within four weeks before surgery. NLR was defined as the absolute neutrophil count divided by the absolute lymphocyte count. Similarly, PLR was calculated by dividing the platelet count by the absolute lymphocyte count. Clinical signs of sepsis and hematologic diseases were not seen in any patient at the time of blood test. Immunosuppressants were not taken by any patient at the time of blood test, except for in the case of one patient who was diagnosed with myeloproliferative disease. Thus, this patient was excluded from our study. This study was approved by the Institutional Review Board of the Catholic Medical College of Korea (reference number: XC20WIDI0102P). Informed consent was waived because of the retrospective nature of this study.

### 2.2. Statistical Analysis

Disease-free survival (DFS) was measured from the date of diagnosis of uterine sarcoma to the date of the first observation of tumor recurrence. It was censored at the time of death or the last follow-up if the patient had no recurrence at that time. Recurrence date was calculated based on the date confirmed by imaging tests (computed tomography, magnetic resonance imaging, abdominal ultrasound, chest X-ray, or PET-CT) performed during the follow-up period. Overall survival (OS) was defined as the time interval from the date of diagnosis to the date of death or the last follow-up. Cutoff values of CA-125 and LDH were determined by the normal reference range in our institutions (0–35 U/mL and 140–271 U/L respectively). Optimal cutoff values of NLR and PLR were determined by the receiver operating characteristic (ROC) curve analysis as the points at which the Yonden index (sensitivity + specificity – 1) values were maximal, using DFS as the endpoint. Patients were categorized into high NLR (≥2.6) and low NLR (<2.6) groups.

NLR and PLR were correlated with clinical and histopathological data by chi-square test or Fisher’s exact test. Associations of clinical/histopathological parameters such as NLR/PLR with DFS/OS were analyzed using Kaplan–Meier curves and compared by the log-rank test. For the multivariate analyses, Cox regression model was used to evaluate effects of prognostic factors. Results are expressed as hazard ratios (HRs). *p* < 0.05 was considered statistically significant. All analyses were performed using SPSS statistical software package, version 22 (IBM SPSS Statistics for Windows, Version 22.0, Armonk, NY, USA). 

## 3. Results

### 3.1. Baseline Characteristics

This study analyzed 99 patients with uterine sarcoma, including 57 (57.6%) patients with LMS, 29 (29.3%) patients with ESS, 7 (7.1%) patients with undifferentiated ESS, and 6 (6.1%) patients with adenosarcoma. Baseline characteristics of patients and tumors are shown in Table 1. The median age at time of diagnosis was 52.1 years (range: 28–81 years). The median follow-up time was 40 months (range: 1–215 months). Numbers (proportions) of patients in stages I, II, III, and IV were 73 (73.7%), 7 (7.1%), 8 (8.1%), and 11 (11.1%), respectively. The study population was stratified into normal body weight (BMI: 18.5–22.9 kg/m^2^) and overweight or obese (BMI ≥ 23 kg/m^2^) according to body mass index (BMI) categories suggested by the World Health Organization for the Asian population. Since NLR and PLR thresholds were not defined, they were distinguished by ROC curve analysis for our patient population. Median NLR level was 3.18 ± 2.18. The optimal cutoff value of NLR was 2.60 for DFS (Area under the curve (AUC): 0.630; 95% CI: 0.511–0.749). Median PLR level was 182.18 ± 74.35. The optimal cutoff value of PLR was 141.12 for DFS (AUC: 0.574; 95% CI: 0.368–0.690). Fifty (50.5%) and 49 (49.5%) patients were stratified into low and high NLR groups, respectively. In terms of medical disease, 25 (25.3%) and 14 (14.1%) of 99 patients had hypertension and diabetes mellitus, respectively. There were no statistically significant differences between the low NLR group and high NLR group. The median tumor size was 9.17 cm (range, 1–29 cm). Thirty-six (36.4%) of 99 patients had a primary lesion larger than 5 cm. More patients in the high NLR group had larger tumor size (89.9% vs. 70.0%, *p* = 0.014) and tumor necrosis (83.7% vs. 66.0%, *p* = 0.007) than in the low NLR group. There was no statistically significant difference in whether lymphadenectomy was performed between the two groups. Interestingly, more patients in the high NLR group experienced recurrence (42.9% vs. 18.0%, *p* = 0.007). All patients underwent primary surgical treatment. Twenty-nine of them underwent pelvic and/or paraaortic lymph node dissection. Fifty-five patients received adjuvant therapy and radiotherapy with or without chemotherapy. 

### 3.2. Univariate and Multivariate Analysis

Univariate analysis revealed that DFS was significantly influenced by advanced stage (III/IV) of sarcoma, subtype of leiomyosarcoma, high mitotic index, preoperative high NLR, and preoperative high PLR. In multivariate analysis after adjusting for baseline parameters, preoperative high PLR (≥141.12) was not a statistically significant prognostic factor for DFS (adjusted HR: 1.90; 95% CI: 0.48–1.57; *p* = 0.363). However, advanced stage (III/IV) (adjusted HR: 2.95; 95% CI: 1.21–7.20; *p* = 0.017), high mitotic index (>15 mitoses/10HPF) (adjusted HR: 2.59; 95% CI: 1.06–6.28; *p* = 0.036), and preoperative high NLR (≥2.60) (adjusted HR: 3.58; 95% CI: 1.42–9.00; *p* = 0.007) retained their prognostic significance for DFS (Table 2).

Univariate analysis revealed that advanced stage (III/IV), subtype of leiomyosarcoma, high mitotic index, nuclear atypia, and preoperative high NLR were significant factors associated with OS. In multivariate analysis after adjusting for baseline parameters, advanced stage (III/IV) (adjusted HR: 3.32; 95% CI: 1.26–8.75; *p* = 0.015), high mitotic index (adjusted HR: 6.01; 95% CI: 1.71–21.20; *p* = 0.005), and high NLR (≥2.60) (adjusted HR: 3.27; 95% CI: 1.06–10.08; *p* = 0.039) retained their prognostic significance for OS. CA-125 and LDH failed to show statistically significant association with DFS or OS (Table 2 and Table 3). Only 53 of 73 patients had LDH data.

**Figure 1:**
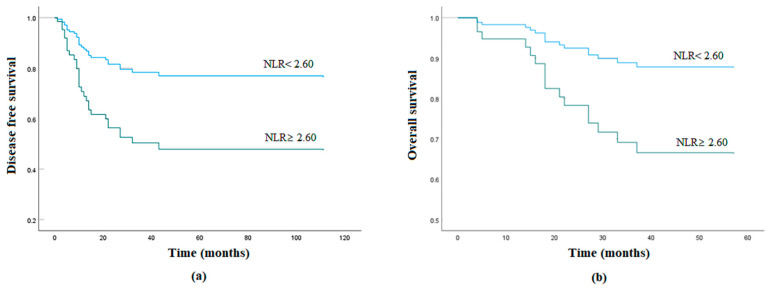
Survival curves of uterine sarcoma according to NLR: (**a**) Kaplan–Meier survival curves for disease-free survival (DFS) of patients with a high NLR and those with a low NLR. Patients with a high NLR had significantly shorter DFS than those with a low NLR (log-rank test, *p* = 0.006). (**b**) Kaplan–Meier survival curves for overall survival (OS) of patients with a high NLR and those with a low NLR. Patients with a high NLR had a significantly shorter OS than those with a low NLR (log-rank test, *p* = 0.045).

Up to June 2020, 30 (30.3%) patients had experienced progression or relapse. Five-year DFS was 63%. A total of 18 (18.2%) deaths occurred. Five-year OS was 73%. Both DFS and OS were significantly lower in the high NLR group than in the low NLR group (DFS: 47.8% vs. 77.6%, *p* = 0.010; OS: 59.4% vs. 86.6%, *p* = 0.047) (Figure 1).

### 3.3. Subgroup Analysis

In subgroup analysis of stage I uterine sarcoma, among 73 patients, 39 (53.4%) and 34 (46.6%) patients were stratified into low and high NLR groups, respectively. More patients in the high NLR group had larger tumor size (≥5 cm) than those in the low NLR group (85.3% vs. 61.5%, *p* = 0.023). Eleven (73.3%) of 15 patients who relapsed had a high NLR (≥2.60) (*p* = 0.020) (Table 4). The high NLR group had a 3.9-fold higher risk of recurrence with worse prognosis than the low NLR group (odds ratio: 4.185; adjusted odds ratio: 3.933; *p* = 0.045) (Table S1, Supplementary Materials). 

## 4. Discussion

Several prognostic factors have been recognized from retrospective data to guide therapeutic decisions for uterine sarcoma patients. Patients with old age, advanced stage, high tumor grade, and high mitotic index have been found to have worse prognosis [13,14,15,16]. However, diagnosis and treatment of uterine sarcoma remain unclear. In the present study, we found that preoperative NLR ratio had clinical usefulness as an independent predictor of recurrence and survival in patients with uterine sarcoma undergoing surgical intervention. Our findings provide a new and valuable clue that disease progression not only can be predicted by histopathological findings such as tumor size, mitotic index, and cancer stage, but also can be determined by host-response factors such as systemic inflammatory response. Inflammation has been reported to be importantly involved in the multistep development of tumorigenesis. It is now considered a hallmark of cancer [17]. Neutrophilia provide a favorable tumor microenvironment for cancer progression by secreting many inflammation mediators such as tumor necrosis factor alpha (TNF-α), vascular endothelia growth factor (VEGF), interleukin-2 (IL-2), interleukin-6 (IL-6), and interleukin-10 (IL-10) [18]. In addition, interactions between the tumor and host cells can induce suppression of immune activities of lymphocytes and activated T-cells. One potential mechanism is that cell-mediated response to tumor infiltration is lymphocyte-dependent [19]. Thus, a low lymphocyte count might be associated with poor prognosis. We have already investigated the clinical impact of NLR as a prognostic factor in malignant pleural effusion (MPE) and a new scoring system that uses NLRs in the serum and MPE of lung cancer patients [20]. 

The usefulness of NLR and PLR in soft tissue sarcoma has been studied extensively. However, there are limitations in applying them to uterine sarcoma. In the area of gynecological cancer, several studies have reported that high NLR is associated with OS and DFS in patients with ovarian, endometrial, or cervical cancer [21,22,23,24]. In uterine sarcoma, some studies have reported that preoperative NLR levels are more useful than CA125 levels for the preoperative differential diagnosis of benign and malignant tumors [25,26]. To the best of our knowledge, there have been only two papers on NLR as a prognostic marker. Kim et al. [9] reported that NLR is not a significant factor for predicting survival of patients with uterine sarcomas. However, their study has included patients with carcinosarcoma, which is currently not classified as sarcoma. Excluding patients with carcinosarcoma, the actual number of patients with uterine sarcoma in their study was only 34. In addition, their follow-up period was short. Recently, Li et al. [10] performed a retrospective study on survival factors in uterine sarcoma patients. Their study identified that histologic type and tumor stage have significant associations with OS in multivariate analysis. An elevated NLR (≥ 3.61) was found to be associated with OS in univariate analysis but not in multivariate analysis. Their study used a different cutoff value (NLR ≥ 3.61) from our study (NLR ≥ 2.60). We reviewed reported results of uterine sarcoma studies using NLR up to the year 2020 (Table 5). Cutoff values of NLR in other studies, including ours, were relatively lower than their cutoff value. Racial variations and personal factors such as age, smoking, and BMI could be associated with cutoff values. Different timing for the collection of blood could also influence outcomes [27]. We calculated NLR by blood test performed within four weeks before surgery. However, they did not mention this in their paper. Differences in proportions according to histopathologic type might have also influenced results. In their study, the most common pathologic type was ESS (55.3%), followed by LMS (29.8%), UUS (7.9%), and adenosarcoma (6.1%) [10]. In our study, LMS had the highest proportion (57.6%). All these factors might have contributed to the difference in results between their study and our study.

Some studies have suggested a new scoring system by combining both NLR and PLR as an independent prognostic factor for OS and DFS [20,28]. Liang et al. [26] defined the combination of NLR and PLR as CNP in soft tissue sarcoma patients. These patients were classified into three CNP groups (both high NLR and high PLR were allocated a score of 2, patients with only one elevated value were allocated a score of 1, patients with both low NLR and low PLR were allocated a score of 0). We applied this to our study and investigated the correlations of CNP with survival and recurrence. As a result, preoperative CNP was found to be a more significant independent prognostic factor than NLR alone, for DFS only (*p* = 0.034) (Tables S2 and S3, Supplementary Materials).

To the best of our knowledge, no study has reported the prognostic relevance of NLR in uterine sarcoma excluding carcinosarcoma and other diseases. In our study, advanced stage, high mitotic index, and high NLR were significantly associated with worse DFS and worse OS. These factors have been considered to be able to indicate worse prognosis and have been well studied as potential poor prognostic factors. In case of advanced stage, aggressive treatment including systemic therapy is strongly recommended even though the role of adjuvant therapy is still a matter of debate. Our data also showed that high NLR was significantly associated with worse DFS and worse OS of not only advanced stage patients, but also patients with stage I uterine sarcoma. In our study, the proportion of patients in stage I was 73.7%. Even with stage I, 11 of 15 patients who relapsed had a high NLR. Previous studies have also reported that high proportions (66.4% and 71.9%) of patients with uterine sarcoma have stage I [12,15]. Treatment options (observation or systemic therapy) for stage I uterine sarcoma including LMS and high grade ESS after surgical resection are controversial and vague according to National Comprehensive Cancer Network (NCCN) guidelines. Our findings suggested that patients in the early stage with high NLR ratio should be considered as having a high risk of poor prognosis. Thus, they should be candidates for more intensive or aggressive adjuvant treatments with close follow-up. Considering that NLR is readily calculated, inexpensive, and universally available in clinical settings from different cell counts in the serum, it will be a valuable indicator. In addition, NLR has been recently highlighted as a prognostic factor for treatment with immunotherapy for advanced cancers such as melanoma, lung cancer, and bladder cancer [29,30,31]. Considering the recent spread of immune therapeutic agents, NLR is expected to be introduced in the treatment of uterine sarcoma in the near future [32]. Therefore, NLR could potentially be a more attractive and ideal marker that might provide valuable additional prognostic information.

This study is somewhat limited. First, all data were retrospectively collected using electronic medical records (EMRs). Thus, clinical and survival comparison might have been influenced by selection bias due to its retrospective nature. Second, there was no defined NLR value for uterine sarcoma patients. Thus, we had to set a NLR cutoff value for our population. Due to the rarity of this disease, the sample sizes for each histologic subtype examined in this study were relatively small. There was a limitation in determining an appropriate cutoff value in the ROC curve. Moreover, our study includes uterine sarcomas (LMS, ESS, UUS, adenosarcoma) with different pathogenesises due to the rarity of the disease. Analyzing the whole study population as subdivided groups with different types limited the statistical power of the current study. Third, preoperative blood count depends on a wide range of factors such as acute or chronic infection, inflammatory disease, and personal lifestyle habits. We considered each patient’s medical condition and medications through the data obtained. It was difficult to elucidate other factors in this retrospective analysis.

## 5. Conclusions

In summary, our study revealed significant associations of preoperative NLR with OS and DFS, suggesting the utility of NLR as a cost-effective and broadly available independent prognostic marker for uterine sarcoma patients. Further studies are needed to confirm our findings and examine the mechanism(s) by which preoperative NLR affects tumor behavior.

## Figures and Tables

**Table 1:** Baseline patient characteristics.

		Total	Low NLR Group	High NLR Group	*p* Value
		*n* = 99	*n* = 50	*n* = 49	
Age (years)	≥60	21 (21.2) ^1^	10 (20.0)	11 (22.4)	0.766
	<60	78 (78.8)	40 (80.0)	38 (77.6)	
BMI (kg/m^2^)	Normal (18.5–22.9)	41 (41.4)	21 (42.0)	20 (40.8)	0.905
	Overweight (≥23)	58 (58.6)	29 (58.0)	29 (59.2)	
Hypertension	Yes	25 (25.3)	14 (28.0)	11 (22.4)	0.525
	No	74 (74.7)	36 (72.0)	38 (77.6)	
Diabetes	Yes	14 (14.1)	8 (16.0)	6 (12.2)	0.592
	No	85 (85.9)	42 (84.0)	43 (87.8)	
Histologic type	LMS	57 (57.6)	27 (54.0)	30 (61.2)	0.433
	ESS	29 (29.3)	17 (34.0)	12 (24.5)	
	UUS	7 (7.1)	2 (4.0)	5 (10.2)	
	Adenosarcoma	6 (6.1)	4 (8.0)	2 (4.1)	
Stage (FIGO)	I	73 (73.7)	39 (78.0)	34 (69.4)	0.801
	II	7 (7.1)	3 (6.0)	4 (8.2)	
	III	8 (8.1)	3 (6.0)	5 (10.2)	
	IV	11 (11.1)	5 (10.0)	6 (12.2)	
Tumor size (cm)	≥5	36 (36.4)	35 (70.0)	44 (89.9)	0.014
	<5	63 (63.6)	15 (30.0)	5 (10.2)	
Mitotic index	≥15	44 (44.4)	20 (40.0)	24 (49.0)	0.369
(MF/10HPF)	<15	55 (55.6)	30 (60.0)	25 (51.0)	
Nuclear atypia	≥severe	20 (20.2)	8 (16.0)	12 (24.5)	0.293
	<severe	79 (79.8)	42 (84.0)	37 (75.5)	
Vascular invasion	Yes	24 (24.2)	12 (24.0)	12 (24.5)	0.955
	No	75 (75.8)	38 (76.0)	37 (75.5)	
Tumor necrosis	Yes	74 (74.7)	33 (66.0)	41 (83.7)	0.043
	No	25 (25.3)	17 (34.0)	8 (16.3)	
Lymphadenectomy	Yes	29 (29.3)	13 (26.0)	16 (32.7)	0.467
	No	70 (70.7)	37 (74.0)	33 (67.3)	
Recurrence	Yes	30 (30.3)	9 (18.0)	21 (42.9)	0.007
	No	69 (69.7)	37 (74.0)	28 (57.1)	
CA-125 (U/mL) ^2^	<35	68 (68.7)	31 (79.5)	23 (67.6)	0.250
	≥35	31 (31.3)	8 (20.5)	34 (32.4)	
LDH (U/L) ^2^	<271	29 (29.3)	8 (20.5)	9 (26.5)	0.548
	≥271	70 (70.7)	31 (79.5)	25 (73.5)	

^1^ Data in parentheses are percentages. Abbreviations: NLR, neutrophil-to-lymphocyte ratio; BMI, body mass index; LMS, leiomyosarcoma; ESS, endometrial stromal sarcoma; UUS, undifferentiated uterine sarcoma; FIGO, International Federation of Gynecology and Obstetrics; MF/10HPF, mitotic figure/10 high-power fields; LDH, lactate dehydrogenase. ^2^ CA-125 and LDH were dichotomized by cutoff of normal value.

**Table 2:** Univariate and multivariate analysis of prognostic factors for disease-free survival (*n* = 99).

	Univariate		Multivariate	
	HR (95% CI)	*p* Value	Adjusted HR (95% CI)	*p* Value
Age (years)		0.141		
≥60	1.74 (0.82–3.82) ^1^			
<60	1.00 (Ref.)			
Histology		0.048		0.498
LMS	2.20 (1.00–4.82)		0.72(0.28–1.86)	
Others	1.00 (Ref.)		1.00 (Ref.)	
Stage (FIGO)		<0.001		0.017
I/II	1.00 (Ref.)		1.00 (Ref.)	
III/IV	4.71 (2.24–9.91)		2.95 (1.21–7.20)	
Tumor size (cm)		0.107		
≥5	2.94 (0.79–10.93)			
<5	1.00 (Ref.)			
Mitotic index (MF/10HPF)		0.004		0.036
≥15	2.98 (1.42–6.26)		2.59 (1.06–6.28)	
<15	1.00 (Ref.)		1.00 (Ref.)	
Nuclear atypia		0.229		
≥severe	1.68 (0.72–3.95)			
<severe	1.00 (Ref.)			
Vascular invasion		0.901		
Yes	1.05 (0.45–2.47)			
No	1.00 (Ref.)			
Tumor necrosis		0.102		
Yes	2.41 (0.84–6.95)			
No	1.00 (Ref.)			
CA-125 (U/mL) ^2^		0.450		
≥35	1.42 (0.57–3.51)			
<35	1.00 (Ref.)			
LDH (U/L) ^2^		0.761		
≥271	1.22 (0.34–4.37)			
<271	1.00 (Ref.)			
NLR		0.009		0.007
≥2.60	3.41 (1.37–8.58)		3.58 (1.42–9.00)	
<2.60	1.00 (Ref.)		1.00 (Ref.)	
PLR		0.009		0.363
≥141.12	4.71 (1.48–14.97)		1.90 (0.48–1.57)	
<141.12	1.00 (Ref.)		1.00 (Ref.)	

^1^ Data in parentheses are percentages. Abbreviations: HR, hazard ratio; CI, confidence interval; FIGO, International Federation of Gynecology and Obstetrics; MF/10HPF, mitotic figure/10 high-power fields; LMS, leiomyosarcoma; LDH, lactate dehydrogenase; NLR, neutrophil-to-lymphocyte ratio; PLR, platelet-to-lymphocyte ratio. ^2^ CA-125 and LDH were dichotomized by cutoff of normal value.

**Table 3:** Univariate and multivariate analysis of prognostic factors for overall survival (*n* = 99).

	Univariate		Multivariate	
	HR (95% CI)	*p* Value	Adjusted HR (95% CI)	*p* Value
Age (years)		0.271		
≥60	1.74 (0.65–4.62) ^1^			
<60	1.00 (Ref.)			
Histology		0.024		0.326
LMS	4.16 (1.20–14.40)		0.47 (0.10–2.13)	
Others	1.00 (Ref.)		1.00 (Ref.)	
Stage (FIGO)		<0.001		0.015
I/II	1.00 (Ref.)		1.00 (Ref.)	
III/IV	5.33 (2.10–13.55)		3.32 (1.26–8.75)	
Tumor size (cm)		0.768		
≥5	1.00 (Ref.)			
<5	0.91 (0.28–2.50)			
Mitotic index (MF/10HPF)		0.010		0.005
≥15	3.63 (1.36–9.60)		6.01 (1.71–21.20)	
<15	1.00 (Ref.)		1.00 (Ref.)	
Nuclear atypia		0.031		0.195
≥severe	2.93 (1.10–7.83)		2.19 (0.67–7.16)	
<severe	1.00 (Ref.)		1.00 (Ref.)	
Vascular invasion		0.578		
Yes	1.00 (Ref.)			
No	0.70 (0.20–2.43)			
Tumor necrosis		0.386		
Yes	1.73 (0.50–5.98)			
No	1.00 (Ref.)			
CA-125 (U/mL) ^2^		0.445		
≥35	1.51 (0.52–4.37)			
<35	1.00 (Ref.)			
LDH (U/L) ^2^		0.902		
≥271	1.90 (0.27–4.48)			
<271	1.00 (Ref.)			
NLR		0.039		0.039
≥2.60	3.25 (1.06–9.97)		3.27 (1.06–10.08)	
<2.60	1.00 (Ref.)		1.00 (Ref.)	
PLR		0.581		
≥141.12	1.37 (0.45–4.25)			
<141.12	1.00 (Ref.)			

^1^ Data in parentheses are percentages. Abbreviations: HR, hazard ratio; CI, confidence interval; FIGO, International Federation of Gynecology and Obstetrics; MF/10HPF, mitotic figure/10 high-power fields; LMS, leiomyosarcoma; LDH, lactate dehydrogenase; NLR, neutrophil-to-lymphocyte ratio; PLR, platelet-to-lymphocyte ratio. ^2^ CA-125 and LDH were dichotomized by cutoff of normal value.

**Table 4:** Baseline patient characteristics in stage I.

		Total	Low NLR Group	High NLR Group	*p* Value
		*n* = 73	*n* = 39	*n* = 34	
Age (years)	≥60	15 (20.5) ^1^	7 (17.9)	8 (23.5)	0.556
	<60	58 (79.5)	32 (82.1)	26 (76.5)	
BMI (kg/m^2^)	Normal (18.5–22.9)	30 (41.1)	17 (43.6)	21 (61.8)	0.643
	Overweight (≥23)	43 (58.9)	22 (56.4)	13 (38.2)	
Hypertension	Yes	18 (24.7)	9 (23.1)	9 (26.5)	0.737
	No	55 (75.3)	30 (76.9)	25 (73.5)	
Diabetes	Yes	11 (15.1)	5 (12.8)	6 (17.6)	0.565
	No	62 (84.9)	34 (87.2)	28 (82.4)	
Histologic type	LMS	37 (50.7)	17 (43.6)	20 (58.8)	0.144
	ESS	23 (31.5)	16 (41.0)	7 (20.6)	
	UUS	7 (9.6)	2 (5.1)	5 (14.7)	
	Adenosarcoma	6 (8.2)	4 (10.3)	2 (5.9)	
Tumor size (cm)	≥5	54 (74.0)	24 (61.5)	29 (85.3)	0.023
	<5	19 (26.0)	15 (38.5)	5 (14.7)	
Mitotic index	≥15	26 (35.6)	12 (30.8)	14 (41.2)	0.354
(MF/10HPF)	<15	47 (64.4)	27 (69.2)	20 (58.8)	
Nuclear atypia	≥severe	13 (17.8)	5 (12.8)	8 (23.5)	0.233
	<severe	60 (82.2)	34 (87.2)	26 (76.5)	
Vascular invasion	Yes	17 (23.3)	9 (23.1)	8 (23.5)	0.964
	No	56 (76.7)	30 (76.9)	26 (76.5)	
Tumor necrosis	Yes	50 (68.5)	23 (59.0)	27 (79.4)	0.061
	No	23 (31.5)	16 (41.0)	7 (20.6)	
Lymphadenectomy	Yes	17 (23.3)	8 (20.5)	9 (26.5)	0.548
	No	56 (76.7)	31 (79.5)	25 (73.5)	
Recurrence	Yes	15 (20.5)	4 (10.3)	11 (32.4)	0.020
	No	58 (79.5)	35 (89.7)	23 (67.6)	
CA-125 (U/mL) ^2^	<35	54 (74.0)	31 (79.5)	23 (67.6)	0.250
	≥35	19 (26.0)	8 (20.5)	11 (32.4)	
LDH (U/L) ^2^	<271	11 (15.1)	6 (23.1)	5 (26.5)	0.682
	≥271	42 (57.5)	20 (76.9)	22 (73.5)	

^1^ Data in parentheses are percentages. Abbreviations: NLR, neutrophil-to-lymphocyte ratio; BMI, body mass index; LMS, leiomyosarcoma; ESS, endometrial stromal sarcoma; UUS, undifferentiated uterine sarcoma; MF/10HPF, mitotic figure/10 high-power fields; LDH, lactate dehydrogenase. ^2^ CA-125 and LDH were dichotomized by cutoff of normal value.

**Table 5:** Overview of uterine sarcoma studies using NLR.

First author	Year	No.	Measurement	NLR Cutoff Value	Outcomes	Histopathology
Kim, H.S. [11]	2010	34	NLR, CA-125	2.12	High NLR as a diagnostic marker	Uterine sarcoma
					High NLR with worse recurrence rate	
Cho, H.Y. [25]	2016	31	NLR	2.1	High NLR as a diagnostic marker	Uterine sarcoma
Li, D. [12]	2020	114	NLR	3.6	High NLR with worse OS	Uterine sarcoma
Zhang, G. [26]	2020	45	NLR, LDH, PLT	2.8	High NLR as a diagnostic marker	LMS
Jeong, M.J. (present study)	2020	99	NLR, PLR, LDH, CA-125	2.6	High NLR with worse OS	Uterine sarcoma
					High NLR with worse DFS	

Abbreviations: NLR, neutrophil-to-lymphocyte ratio; OS, overall survival; LDH, lactate dehydrogenase; PLT, platelet; LMS, leiomyosarcoma; PLR, platelet-to-lymphocyte ratio; DFS, disease-free survival.

## Supplementary Materials

The following are available online at https://www.mdpi.com/2077-0383/9/9/2898/s1, Table S1: The relationship between NLR, PLR level and survival/recurrence rate in stage I uterine sarcoma, Table S2: Baseline characteristics according to Combined NLR and PLR (CNP score), Table S3: Univariate and multivariate analysis of prognostic factors for the Disease-Free Survival.
